# Implementation of interventions to maintain and promote the functional mobility of nursing home residents – a scoping review

**DOI:** 10.1186/s12877-023-04213-5

**Published:** 2023-09-26

**Authors:** Tina Quasdorf, Christina Manietta, Mike Rommerskirch-Manietta, Jana Isabelle Braunwarth, Christin Roßmann, Martina Roes

**Affiliations:** 1 School of Health Science, Institute of Nursing, ZHAW Zürich University of Applied Science, Winterthur, Switzerland; 2 Deutsches Zentrum für Neurodegenerative Erkrankungen e.V. (DZNE), Standort Witten, Witten, Germany; 3 Fakultät für Gesundheit, Universität Witten/Herdecke, Witten, Germany; 4 Bundeszentrale für gesundheitliche Aufklärung (BZgA), Köln, Germany

**Keywords:** Long-term care, Functional mobility, Implementation, Implementation strategies, Barriers, Facilitators

## Abstract

**Background:**

To provide an overview of the available evidence on the implementation of direct and capacity-building interventions to promote and maintain the functional mobility of nursing home residents.

**Methods:**

We conducted a scoping review following the methodological guidance for the conduct of scoping reviews as described by the Joanna Briggs Institute. We searched for studies in MEDLINE (via PubMed) and CINAHL (via EBSCO). We conducted a qualitative content analysis of the included studies with deductive categories based on the Consolidated Framework for Implementation Research (CFIR).

**Results:**

Ultimately, we included 8 studies on direct interventions, 6 studies on capacity-building interventions, and 2 studies on both types of interventions in our review. Seven studies provided evidence on implementation strategies comprising discrete as well as multifaceted, multilevel strategies. Most of the studies did not systematically evaluate the strategies but remained at a descriptive level. All 16 studies provided evidence of influencing factors. We identified 32 of the 37 influencing factors of the CFIR. The five most frequent influencing factors were *available resources* (*n* = 14), *access to knowledge and information* (*n* = 12), *patient needs and resources* (*n* = 10), *knowledge and beliefs about the intervention* (*n* = 10) and *compatibility* (*n* = 9).

**Conclusions:**

The available evidence on the implementation of functional mobility interventions in nursing homes is rather limited. This emphasizes the need for further research. Regarding implementation strategies, the systematic evaluation and further development of the reported promising approaches might be a starting point.

**Supplementary Information:**

The online version contains supplementary material available at 10.1186/s12877-023-04213-5.

## Background

Functional mobility is an essential precondition for independence [1–3] and autonomy [3, 4] in many areas of life. It is important for social participation [5, 6], quality of life [7–11] and subjective well-being [10] as well as for preventing the decline of physical health [12]. In turn, immobility leads to negative consequences, such as negative health outcomes [12–14], increased risk of falls [9, 12, 15], and increased healthcare utilization [12]. Apart from cognitive disorders, immobility is one of the main causes of care dependency [12, 16].

Most of these negative consequences frequently occur for nursing home (NH) residents, and thus, the decline of physical performance is also evidently associated with them [17, 18]. Given this situation, maintaining and enhancing functional mobility are important factors for preventing health restrictions and increasing the care dependency of NH residents. Thus, maintaining and enhancing functional mobility are pivotal tasks of professional care in NHs [16, 19].

Many interventions are available to enhance functional mobility and to prevent the functional decline of residents. In addition to interventions that focus directly on the behavior of the person whose functional mobility is to be maintained or enhanced (e.g., exercise programs, walking programs), there is an increased awareness that interventions that aim to improve organizational capacity to promote the functional mobility of residents are needed as well (e.g., education for nursing staff or environmental changes) [20]. Either type of mobility intervention tends to be quite complex and to comprise a variety of components [21–23].

For the development of such complex interventions, it is strongly recommended not only that the outcomes of the intervention be evaluated but also that the process of implementing the intervention be considered [24–27]. This is especially recommended since the benefit depends not only on the effectiveness of the intervention itself but also on its successful implementation in real-life settings [27]. Multiple factors can influence such implementation processes [28, 29] and need to be addressed by tailored implementation strategies [30].

To ensure the effective and sustainable implementation of mobility interventions, these influencing factors need to be considered and implementation strategies need to be systematically investigated. However, to date, such implementation aspects of mobility interventions have only marginally been investigated, and to our knowledge, a comprehensive and systematic overview of evidence is missing.

To close this research gap, we conducted a scoping review with the objective to identify and descriptively summarize the available evidence on implementation strategies and influencing factors for the implementation of interventions to promote and maintain the functional mobility of NH residents. Considering (A) direct interventions as well as (B) organizational capacity-building interventions we addressed the following two research questions:

- What strategies for the implementation of **(A) direct interventions** and **(B) organizational capacity-building interventions** to promote and maintain the functional mobility of NH residents have been investigated?
- What factors influence the implementation of **(A) direct interventions** and **(B) organizational capacity-building interventions** to promote and maintain the functional mobility of NH residents?

## Methods

Scoping reviews are especially recommended to explore the extent of the available literature and to map and summarize the evidence in a given field [31]. Thus, to conduct this review, we followed the methodological guidance for the conduct of scoping reviews as described by the Joanna Briggs Institute [31, 32]. Accordingly, we used the Preferred Reporting Items for Systematic reviews and Meta-Analyses extension for Scoping Reviews (PRISMA-ScR)[33] for the reporting of the review (Supplementary Table S1). Furthermore, we developed an internal review protocol to guide the process.

### Literature search

We developed two separate search strategies addressing the two different types of mobility interventions (A, B). For the development of the search strategies, we adapted the ‘Population, Concept of interest, Context (PCC)’ mnemonic [31] and clustered our search terms accordingly: population = nursing home (1); concept of interest = implementation strategies and influencing factors for the implementation (2); and context = interventions to promote and maintain the functional mobility (3). We developed a set of search terms for each PCC element, which we adapted after an initial explorative search. We combined the PCC elements into two search strategies. While we differentiated element 3 (context) with regard to the type of mobility intervention (A, B), elements 1 and 2 remained the same for both search strategies. Additionally, we used key publications to identify free search terms and indexing words. The search strategies were developed by two reviewers (TQ, CM) and were then reviewed and discussed within the project team (TQ, CM, MRM, JIB, MR) and with the Bundeszentrale für gesundheitliche Aufklärung (BZgA)(CR) based on the Peer Review of Electronic Search Strategies [34]. Searches were conducted in the following electronic databases: MEDLINE (via PubMed) and CINAHL (via EBSCO). The search strategy was first developed for MEDLINE (Supplementary Table S2) and then adjusted for CINAHL with RefHunter V.5.0 [35]. The searches were conducted in November 2020. Additionally, we performed backward and forward citation tracking via reference lists of the included studies.

### Study selection

We defined the inclusion criteria based on the research aims and questions and clustered them according to the PCC mnemonic [36]. Additionally, we defined criteria for the type of evidence and language (Table 1).

**Table 1:** Inclusion criteria

Criteria	Definition
Population	Nursing home (incl. NH residents, NH staff, NH environment)
Concept	Implementation of mobility interventions: – strategies for the implementation **AND/OR** – factors influencing the implementation
Context	Interventions to maintain or promote mobility: – direct interventions (A) = interventions that focus directly on the behavior of the resident, or – organizational capacity-building interventions (B) = interventions for changing the ◦ ecological, ◦ social, ◦ organizational-cultural, and ◦ technical-material living conditions in the institutional and social context with the aim of maintaining or improving various health determinants of the people living in these settings [37, 38]
Types of evidence	Any kind of study
Types of sources	Peer-reviewed articles and gray literature in the form of study reports
Languages	English and German

References identified through our literature search were imported into Covidence software [39] and automatically checked for duplicates. Titles and abstracts of the remaining references were independently screened against the inclusion criteria by two reviewers (TQ, CM). Divergent ratings were discussed between the two reviewers, and in case of no consensus, the respective references were discussed with selected coauthors (MRM, JIB, MR). For the full-text screening, we applied the same strategy. Covidence software [39] was used for both screening steps.

### Data extraction and management

We adapted the Joanna Briggs Institute template for scoping reviews [36] for data extraction. According to the procedures described by the Joanna Briggs Institute [36], we developed the final data extraction template in an iterative process. This means that we validated and adjusted the initial template, while the first studies were extracted until all relevant data were represented with the template. Finally, the following data were extracted from all studies: study name, authors, year of publication, country, study aim, study design, methodological/ theoretical approach, methods, sites, study population, mobility intervention (incl. target population), implementation outcomes, evidence on 1) implementation strategies and 2) factors influencing implementation. Data extraction was performed by one reviewer (TQ) and then cross-checked by a second reviewer (CM).

### Data analysis and synthesis

The identified implementation strategies are summarized descriptively.

To analyze the influencing factors of implementation in the included studies, we conducted a qualitative content analysis [40]. We derived the initial deductive categories from the Consolidated Framework for Implementation Research (CFIR) [29] (Supplementary Table 3). Using MAXQDA V. 2020.2.0 [41], the included studies were coded by one reviewer (TQ or CM), and each was cross-checked by the other reviewer. Both reviewers discussed divergent ratings, and recoding was undertaken if necessary.

### Stakeholder conference

We presented the findings to and discussed them with CR (BZgA) and five stakeholders from different welfare organizations in long-term care to confirm and refine our interpretations. The stakeholders were managers and project managers, who were responsible for organizational development and quality improvement within their organizations on a regular basis.

## Results

**Fig. 1:**
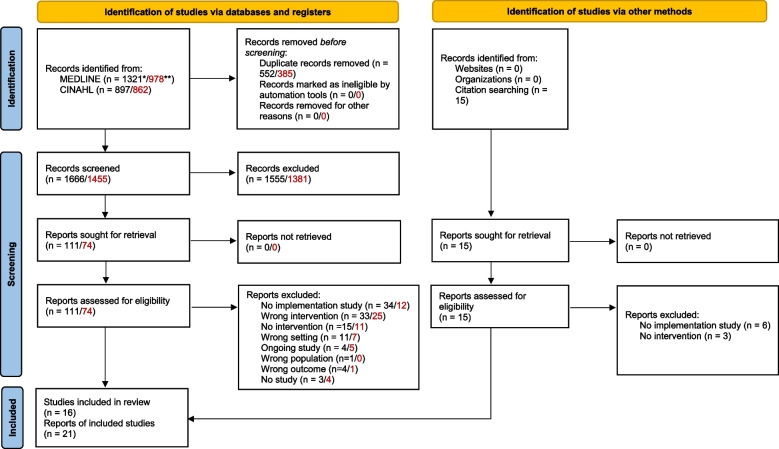
PRISMA 2020 flow diagram for new systematic reviews, which included searches of databases, registers and other sources [63]. *Numbers for search strategy A (direct interventions) are presented in black/on the left side throughout the flow diagram. **Numbers for search strategy B (capacity-building interventions) are presented in red/on the right side throughout the flow diagram

Through the electronic database searches, we identified a total of 2218 (A) and 1841 (B) records. After deduplication, we screened 1666 (A)/1453 (B) titles and abstracts for relevance, which resulted in the screening of 111 (A)/72 (B) full texts. Finally, we included 16 studies reported in 21 reports [42–62] in the review (Fig. 1).

### Study characteristics

Table 2 provides an overview of the included studies. The studies were from different countries: Canada (*n* = 3) [42, 43, 45, 46, 51], Australia (*n* = 3)[47, 49, 50], the Netherlands (*n* = 3) [57, 60, 61], the UK (*n* = 2) [52, 56, 58], the USA (*n* = 3) [44, 53, 59], and Germany (*n* = 2) [48, 62]. They were published between 2006 and 2020. A variety of designs were used within the studies and their sub-studies, including a qualitative design (*n* = 5)[43, 49, 50, 53, 59], mixed methods (*n* = 3) [56, 57, 61], process/scientific evaluation (*n* = 3) [46, 48, 60], case study (*n* = 2) [47, 58], quasi-experimental pilot study (*n* = 1) [51], cluster randomized trial (*n* = 2)[45, 62], quality improvement project (*n* = 1) [44], cohort study (*n* = 1) [52], and quantitative pre-post-design (*n* = 1) [42].

**Table 2:** Overview of the included studies

General information		Study aims, design & methods	Participants	Mobility intervention	Evidence^a^
**(A) direct interventions to promote and maintain the mobility of nursing home residents**					
**PED Project**	Brett et al. 2018 [49] Australia	**Aim:** to gain insights into ▪ staff & family carers’ views, attitude, opinions of physical exercise ▪ the feasibility of conducting a physical exercise intervention for individuals living with dementia in nursing homes **Study design:** ▪ qualitative study (following an effectiveness RCT) **Methodological/theoretical approach:** ▪ interpretive description **Methods:** ▪ semistructured interviews	**Sites:** ▪ nursing homes (*n* = 2) **Study population:** ▪ staff (*n* = 10) ◦ registered nurses (*n* = 3) ◦ lifestyle & recreational officers (*n* = 3) ◦ direct care workers (*n* = 2) ◦ physical therapy assistant (*n* = 1) ◦ occupational health and safety representative (*n* = 1) ▪ family carers (*n* = 9)	**Intervention:** ▪ physical exercise **Target population:** ▪ individuals living with dementia in nursing homes	2
	Gomaa et al. 2020 [50] Australia	**Aim:** to elucidate the benefits, challenges, and facilitators/barriers to the implementation of music-cued therapeutic dancing in residential aged care **Study design:** ▪ qualitative approach **Methodological/theoretical approach:** ▪ realist theoretical framework **Methods:** ▪ semistructured interviews	**Sites:** ▪ 60-bed high-dependency residential aged care setting (*n* = 1) **Study population:** ▪ dance instructor (*n* = 1) ▪ music therapist (*n* = 1) ▪ physiotherapists (*n* = 2) ▪ class assistant (*n* = 1) ▪ facility manager (*n* = 1) ▪ lifestyle program coordinator (*n* = 1)	**Intervention:** ▪ music-cued dancing: ◦ 12 sessions over 8 weeks with 5–8 participants ◦ 30-min movement component + 5-min warm up & cool down ◦ dancing movements/steps matched to the abilities/preferences of residents ◦ genres: tap dance, tango, salsa, modern, jazz, creative dance **Target population:** ▪ frail older people living with or without dementia in residential aged care	2
	Horn et al. 2019 [48] Germany	**Aim:** to identify and analyze ▪ facilitators to and barriers of the implementation of a physical activity program ▪ challenges to its continuation **Study design:** • scientific evaluation **Methodological/theoretical approach:** • not reported **Methods:** • semistructured/problem-centered interviews, observations, analysis of documents, standardized collection of health data, standardized data collection of structural characteristics and culture of the participating organizations	**Sites:** • inpatient care facilities** **Study population:** ▪ management** ▪ quality management** ▪ nursing staff** ▪ trainers**	**Intervention:** ▪ ‘Lübeck Worlds of Movement Model’ ◦ group intervention (6–12 participants ◦ 2 sessions lasting 60 min per week ◦ trained group leader ◦ close to everyday life exercises ◦ accompanying individual training program based on a group program **Target population:** ▪ nursing home residents	2
**Aging and new media**	Juul et al. 2019 [47] Australia	**Aim:** to investigate the role of touchscreen technology in facilitating increased physical activity and stimulating social interaction in RACFs in order to decrease social & physical inactivity **Study design:** • case study design **Methodological/theoretical approach:** • qualitative ethnographic fieldwork **Methods:** • observation, targeted informal conversations, video ethnography, in-depths semistructured interviews	**Sites:** • residential aged care facility (*n* = 1) **Study population:** • residents** • staff** • visitors (residents’ family & friends)**	**Intervention:** • Touchscreen technology ◦ multiple user interactions using the device at the same time ◦ 165-cm interactive portable touch screen ◦ interactive physical activity video – sitdance -uploaded and projected onto the screen ◦ sitdance: seated dance tutorial specially designed for older people; not only an exercise program but also designed to support older people’s memory & encourage social interaction **Target population:** ▪ residents	2
	Kazana & Pencak Murphy 2018 [44] USA	**Aim:** to describe the development of a walking program including process, outcomes, and lessons learned at the LTC facility that was the project site **Study design:** • quality improvement project **Methodological/theoretical approach:** • not reported **Methods:** • logs, reports, chart review, observations	**Sites:** • inner-city skilled nursing and living center (*n* = 1) **Study population:** • residents (*n* = 13)	**Intervention:** • walking program ◦ based on individual walking goals ◦ walking activity provided at least 5 x/week ◦ assigned & provided by certified nursing assistants & restorative staff **Target population:** residents	1 & 2
	Slaughter & Estabrooks 2013 [51] Canada	**Aim:** to assess the effect of the sit-to-stand activity (evidence) on the mobility outcomes of nursing home residents, the effect of an audit-and feedback intervention (facilitation) on the uptake of the sit-to-stand activity by healthcare aides, and the contextual factors influencing the uptake of the activity (context) **Study design:** • quasi-experimental pilot study **Methodological/theoretical approach:** • Promoting Action on Research Implementation in Health Services (PARIHS) framework **Methods:** • documentation of resident performance of the sit-to-stand activity, interview-based survey, Alberta Context Tool (56-item survey instrument)	**Sites:** • nursing homes (*n* = 2) **Study population:** • residents (*n* = 45) • health care aides (*n* = 56)	**Intervention:** ▪ ‘Sit‐to‐stand mobility activity’: ◦ health care aides prompt residents to repeatedly stand up & sit down on 4 occasions during daily functional activities (2 × per day & evening shift) ◦ number of repetitions vary according to residents’ ability and fatigue ◦ activity is to be integrated into usual care routines ◦ timing & location at the discretion of the health care aide **Target population:** residents	1 & 2
**MOVE study**	Slaughter et al. 2018 [46] Canada Study protocol: Slaughter et al. 2011 [54] Canada	**Aim:** to evaluate the processes & perceived outcomes of 8 knowledge translation interventions in a study that introduced a mobility innovation into the daily care practices of healthcare aides **Study design:** • mixed methods process evaluation **Methodological/theoretical approach:** • not reported **Methods:** • interviews, focus groups, ranking exercise	**Sites:** • long‐term care facilities (*n* = 3) **Study population:** • healthcare aides (*n* = 27) • leaders (*n* = 4)	**Intervention:** ▪ ‘Sit‐to‐stand mobility activity’: ◦ health care aides prompt residents to repeatedly stand up & sit down on 4 occasions during daily functional activities (2 × per day & evening shift) ◦ number of repetitions vary according to residents’ ability and fatigue ◦ activity is to be integrated into usual care routines ◦ timing & location at the discretion of the health care aide **Target population:** ▪ residents	1 & 2
**START study**	Slaughter, et al. 2020 [45] Canada Study protocol: Slaughter et al. 2013 [55] Canada	**Aim:** • to determine the effectiveness of a novel knowledge translation intervention, the peer reminder, compared to a standard paper reminder intervention • to examine reminder intensity and reminder frequency on the 1- year sustainability of care aides completing and documenting a mobility intervention with residents living in care facilities **Study design:** • cluster randomized controlled trial, using a stratified factorial design **Methodological/theoretical approach:** • not reported **Methods:** • recording of the conduct of the sit- to-stand activity; questionnaires to assess frequency, duration, content, deviations, modifications of the reminders as well as additional reminders; direct observation to assess the fidelity of the paper reminder intervention	**Sites:** • long-term care facilities (*n* = 8) • assisted living facilities (*n* = 15) **Study population:** • residents (*n* = 349) • care aides (peer reminder) (*n* = 54)	**Intervention:** ▪ ‘Sit‐to‐stand mobility activity’: ◦ health care aides prompt residents to repeatedly stand up & sit down on 4 occasions during daily functional activities (2 × per day & evening shift) ◦ number of repetitions vary according to residents’ ability and fatigue ◦ activity is to be integrated into usual care routines ◦ timing & location at the discretion of the health care aide **Target population:** ▪ residents	1 & 2
	Kagwa et al. 2018 [43] Canada	**Aim:** • to explore the experience of healthcare aides encouraging residents living in residential care facilities to complete the sit-to-stand activity • to identify the strategies healthcare aides use to integrate this activity into their work routines **Study design:** • qualitative substudy of the START study (cluster randomized controlled trial) **Methodological/theoretical approach:** • not reported **Methods:** • semistructured interviews	**Sites:** • long-term care facilities (*n* = 2) • assisted living facilities (*n* = 5) **Study population:** • health care aides (*n* = 7)	**Intervention:** ▪ ‘Sit‐to‐stand mobility activity’: ◦ health care aides prompt residents to repeatedly stand up & sit down on 4 occasions during daily functional activities (2 × per day & evening shift) ◦ number of repetitions vary according to residents’ ability and fatigue ◦ activity is to be integrated into usual care routines ◦ timing & location at the discretion of the health care aide **Target population:** residents	2
	Tworek et al. 2019 [42] Canada	**Aim:** to examine the effect of two knowledge translation interventions, informal walkabouts and documentation information sessions, on supporting initial care aide adoption of a new evidence-based practice, the sit-to-stand activity **Study design:** • quantitative pre-post-substudy of the START study (cluster randomized controlled trial) **Methodological/theoretical approach:** • not reported **Methods:** • analysis of documentation sheets/resident charts	**Sites:** • long-term care facilities (*n* = 8) • assisted living facilities (*n* = 15) **Study population:** • residents (*n* = 227)	**Intervention:** ▪ ‘Sit‐to‐stand mobility activity’: ◦ health care aides prompt residents to repeatedly stand up & sit down on 4 occasions during daily functional activities (2 × per day & evening shift) ◦ number of repetitions vary according to residents’ ability and fatigue ◦ activity is to be integrated into usual care routines ◦ timing & location at the discretion of the health care aide **Target population:** ▪ residents	1
**(B) organizational capacity-building interventions to promote and maintain the mobility of nursing home residents**					
	Den Ouden, et al. 2019 [61] Netherlands	**Aim:** to examine the feasibility of DAIly NURSE and a nursing intervention to encourage nursing home residents’ daily activities and independence **Study design:** • feasibility testing using a mixed-methods design **Methodological/theoretical approach:** • framework of Saunders et al. [64] **Methods:** • self-administered evaluation questionnaires, attendance lists, notes of workshops, focus group interview, MAINtAIN questionnaire [65], background data on nursing home residents and nursing staff	**Sites:** • nursing homes (*n* = 2) **Study population:** • residents (*n* = 20) • nursing staff (*n* = 13)	**Intervention:** ▪ Daily Activities and Independence by NURsing Staff Encouragement” (DAIly NURSE) ◦ multicomponent nursing intervention ◦ aims to change nursing staff behavior in a way that nursing home residents are encouraged & supported to perform their daily activities as independently as possible ◦ components: education, coaching-on-the-job and policy **Target population:** ▪ nursing staff	2
**Projekt ExMo**	Görres et al. 2016 [62] Germany	**Aim:** to evaluate the exemplary implementation of the draft expert standard “Maintenance and promotion of mobility in care” **Study design:** • cluster randomized trial & observational study **Methodological/theoretical approach:** • not reported **Methods:** • nursing records, staff survey, questionnaires, process documentation, structural data, cost data, telephone interviews	**Sites:** • nursing homes (*n* = 33) • semiresidential care/day care (*n* = 6) • home care (*n* = 6) **Study population:** • residents** • nursing staff **	**Intervention:** ▪ Intervention group A: education session on implementing the German national mobility expert standard ▪ Intervention group B: same intervention as group A in addition to an explicit mobility training for promoting the mobility of the residents **Target population:** ▪ care services ▪ care staff ▪ residents	1&2
	Henskens et al. 2017 [60] Netherlands	**Aim:** • to test the effect of movement-oriented restorative care (MRC) among NH residents with moderate to severe dementia • additionally, data was collected regarding the degree of implementation, and the barriers to the implementation process **Study design:** • process evaluation within a quasi-experimental study **Methodological/theoretical approach:** • theoretical elements from previous studies **Methods:** • only process evaluation: questionnaires, focus groups	**Sites:** • locations of a long-term care organization (*n* = 2) **Study population:** • residents (*n* = 61) • professionals (*n* = 12) ◦ nurses (*n* = 3) ◦ activity supervisors (*n* = 3) ◦ heads of departments (*n* = 2) ◦ physiotherapist (*n* = 1) ◦ occupational therapist (*n* = 1) ◦ ‘ambassadors’ (*n* = 2)	**Intervention:** ▪ ‘Movement-oriented Restorative Care’ (MRC) ◦ derived from the concept of function-focused care = multidisciplinary approach toward nursing home dementia care that focuses on stimulating physical activity & independent functioning ◦ key elements: educating nursing staff and families, establishing goals with each resident, administering process evaluations to determine the extent to which MRC was implemented as intended **Target population:** ▪ nursing home residents with dementia	2
	Kuk et al. 2017 [57] Netherlands	**Aim:** to evaluate the feasibility of the TIP-toolbox, an instrument developed to support nursing staff step-by-step in implementing an innovation in nursing homes in order to further improve the toolbox for the needs of its end-users **Study design:** • feasibility study with a mixed-methods design **Methodological/theoretical approach:** • Medical Research Council (MRC) guidance for process evaluation of complex interventions [25] • work by Saunders et al. [64] **Methods:** • questionnaires, telephone interviews, participant observations, and focus group interviews	**Sites:** • nursing homes (embedded in the Living Lab in Aging and Long-Term Care)(*n* = 3) **Study population:** • registered nurses (*n* = 12) ◦ vocationally trained (n = 9) ◦ bachelor educated (*n* = 3)	**Intervention:** ▪ ‘Translating Innovations into Practice-toolbox’ (TIP-toolbox) ◦ supports nursing staff in developing a structured and tailored implementation plan to sustainably implement an innovation in a specific setting ◦ based on implementation of change model by Grol et al. [66] ◦ paper booklet or PDF format, supplemented with electronic tools ◦ focus of this feasibility study: implementation of innovations related to the promotion of functional activity **Target population:** ◦ nursing staff (TIP-toolbox)	1 & 2
	Resnick et al. 2006 USA [53]	**Aim:** • to explore with nursing assistants their feelings and experiences related to restorative care nursing activities after participating in the implementation of a restorative care program **Study design:** • qualitative study **Methodological/theoretical approach:** • not reported **Methods:** • focus groups	**Sites:** • nursing home (*n* = 1) **Study population:** • nursing assistants (*n* = 13)	**Intervention:** • Res-Care Pilot Intervention ◦ 2-tiered intervention focused on motivating nursing assistants to engage in restorative care activities and teaching them how to motivate the residents to do likewise **Target population:** nursing assistants	2
	Resnick et al. 2008 [59] USA	**Aim:** • to explore nursing assistants’ experience in participating in a restorative care intervention study **Study design:** • qualitative study **Methodological/theoretical approach:** • not reported **Methods:** • focus groups	**Sites:** • nursing homes (*n* = 6) **Study population:** • nursing assistants (*n* = 93)	**Intervention:** • Res-Care Intervention ◦ 2-tiered intervention focused on motivating nursing assistants to engage in restorative care activities and teaching them how to motivate the residents to do likewise **Target population:** • nursing assistants	2
**(A) direct interventions & (B) organizational capacity building interventions to promote and maintain the mobility of nursing home residents**					
**OPERA study**	Ellard et al. 2014 [56] UK	**Aim:** to explore potential explanations for the lack of effect of the intervention in the OPERA cluster randomized trial **Study design:** ▪ mixed methods approach **Methodological/theoretical approach:** ▪ phenomenological approach for all qualitative work **Methods:** ▪ quantitative data from all the study homes (organizational characteristics, process data questionnaires) ▪ quantitative and qualitative data from a purposive sample of eight case study homes (observations, interviews, focus groups, checklists)	**Sites:** ▪ care homes (*n* = 78) **Study population:** ▪ residents^b^ ▪ care staff^b^ ▪ managers^b^ ▪ OPERA research staff^b^	**Direct mobility intervention:** ▪ twice-weekly, moderate intensity, progressive group exercise sessions led by a physiotherapist **Target population:** ▪ care home residents **Organizational capacity building intervention** ▪ activities aimed at changing the culture of the homes so that residents would be supported and encouraged to be more active: ◦ physiotherapy assessments and exercise prescriptions for all residents ◦ advice for staff on ways to safely increase the mobility of the residents ◦ provision of simple aids to maximize individuals’ mobility ◦ formal care home staff training on recognizing depression and the potential importance of promoting physical activity in residents **Target population:** ▪ care home staff	2
	Finnegan et al. 2015 [52] UK	**Aim:** to determine individual and ‘home-level’ predictors of attendance at physiotherapy led exercise groups **Study design:** ▪ cohort study nested into a cluster-randomized controlled trial (OPERA study) **Methodological/theoretical approach:** ▪ not reported **Methods:** ▪ used quantitative data from the RCT: attendance rates & predictive factors at resident level (e.g., number of chronic conditions, depression, lower limb function, social engagement, fear of falling) & and on home level	**Sites:** ▪ residential homes (*n* = 25) ▪ nursing homes (*n* = 9) **Study population:** ▪ residents (*n* = 428)	**Direct mobility intervention:** ▪ twice-weekly, lower level or moderate intensity, progressive group exercise sessions led by a physiotherapist **Target population:** ▪ care home residents	2
	Turpie et al. 2017 [58] Scotland, UK	**Aim:** • to report on the implementation of a physical activity (PA) scheme – Let’s Motivate (LM) • to provide an insight into the different factors which might contribute to its success and further **Study design:** • qualitative case study design **Methodological/theoretical approach:** • a series of theoretical resources **Methods:** • one-to-one semistructured interviews	**Sites:** • private care homes (*n* = 2) **Study population:** • key staff from each care home (*n* = 6) ◦ support workers (*n* = 5) ◦ senior support worker (*n* = 1) • regional manager of the care homes (*n* = 1) • LM training instructor (*n* = 1)	**Intervention:** • Let’s Motivate (LM) initiative ◦ aims to improve the health, wellbeing and quality of life of older adults in care homes by developing opportunities for them to be more physically active ◦ aspires to transform the very nature of the care home “setting”, making it more conducive to physical activity ◦ recognizes staff as a key resource ◦ staff training as key element of the initiative ◦ simple and undemanding activities provided to residents **Target population:** care home staff (training), residents (physical activity)	2

^a^evidence reported with regard to the review research questions: 1 = implementation strategies, 2 = barriers/facilitators to implementation; ^b^(n) not or not comprehensibly reported

### Description of the implementation strategies

Seven studies [42, 44–46, 51, 57, 62] of the 16 included studies provided evidence on implementation strategies. Different implementation strategies such as staff education/trainings/information [42, 44, 55, 62], different types of reminders [45, 46, 51], audit and feedback interventions [44, 46, 51, 62], guiding coalitions [44, 62], assessment of environmental/influencing factors [44, 57], and development of individual implementation strategies [57] were described. All studies combined at least two different implementation strategies. Implementation outcomes such as fidelity [45, 57], sustainability [45], adherence [44], dose [57], context [57], satisfaction [57], complexity [57], adaptations [57], and intervention uptake [42, 51] were used to examine the effect or the feasibility of the implementation strategies or to evaluate the implementation.

Table 3 gives an overview of the studies that assessed implementation strategies and their results related to the implementation strategies.

**Table 3:** Overview of the implementation strategies identified within the included studies

Study	Mobility intervention	Implementation study	Implementation strategies	Study results regarding implementation
**(A) direct interventions to promote and maintain the mobility of nursing home residents**				
Slaughter & Estabrooks 2013 [51]	Sit-to-stand activity	Aim*: to examine the effect of an audit-and-feedback intervention on the uptake of the sit-to-stand activity by healthcare aides **Study design:** quasi-experimental pilot study **Implementation outcome:** healthcare aides’ intervention uptake **Methods:** documentation flowsheets and a survey-based measure	**Site 1:** • education sessions for healthcare aides • paper-based reminders (bedside stickers and a conference room poster) • audit-and-feedback intervention (including summary of the resident’s mobility outcomes in a poster & outcome presentation to the Director of Care, healthcare aids and other staff) **Site 2:** • education sessions for healthcare aides • paper-based reminders (bedside stickers and a conference room poster)	• The audit and feedback intervention was associated with increased intervention uptake over time • Uptake increased in site 1, where the initial uptake was weak. In contrast, the uptake in site 2 was higher in the beginning and remained relatively constant
**MOVE study** Slaughter et al. 2018 [46]	Sit-to-stand activity	**Aim:** to examine the perceived effectiveness of eight knowledge translation interventions to implement the sit-to-stand activity **Study design:** mixed methods process evaluation **Implementation outcome:** effectiveness of knowledge translation interventions perceived by healthcare aides and leaders **Methods:** interviews, focus groups, ranking	Knowledge translation interventions 1. flowsheet annotation and informal discussions 2. paper reminder system 3. focus group 4. focus group poster and strategies sheet 5. flowsheet follow-up discussion 6. leader endorsement 7. healthcare aid champions 8. audit and feedback poster	• Reminders, followed by discussion groups, were perceived as most effective by leaders and healthcare aids to sustain practice change • Champions were perceived as least effective • Leaders rated focus groups and audit and feedback posters as the knowledge translation interventions most difficult to realize
**START study**				
Slaughter, et al. 2020 [45]	Sit-to-stand activity	**Aim:** to compare the effectiveness of four different reminder interventions to sustain the sit-to-stand activity **Study design:** cRCT, using a stratified factorial design **Implementation outcome:** fidelity, sustainability **Methods:** flowsheets, questionnaires, observations	**Group 1** • monthly socially based peer reminders **Group 2** • quarterly socially based peer reminders **Group 3** • monthly paper-based reminders **Group 4** • quarterly paper-based reminders	• Paper reminders were implemented with high fidelity (91.5% per protocol), while the peer reminders were implemented with moderate to poor fidelity (monthly 81.0%/ quarterly 51.7% per protocol) • The average sustainability after 12 months was about twice as high in the monthly socially based peer reminder group than in the others
Tworek et al. 2019 [42]	Sit-to-stand activity	**Aim:** to examine the effect of two knowledge translation interventions on supporting initial care aide adoption of the sit-to-stand activity **Study design:** quantitative pre-post-substudy of the START study (cluster randomized controlled trial) **Implementation outcome:** intervention uptake **Methods:** documentation sheets	Knowledge translation intervention during the first 4 month: • two Informal walkabouts with care aids (i.e. spontaneous short meetings in the hall) • two documentation information sessions with care aids (to clarify the flow charts used for documentation)	• After adjusting for age, sex, dementia status, location, and mobility, an increase in uptake of the sit-to-stand activity was observed over the 4-month period (day shift: 5.3% mean increase, evening shift: 6.1% mean increase) • The site size had a significant effect on the outcome (12.6% (SE = .07) increase over small sites and a 18.2% (SE = .05) increase over large sites)
Kazana & Pencak Murphy 2018 [44]	Walking program	**Aim:** to develop, implement, and evaluate a patient-centered walking program **Study design:** quality improvement project **Implementation outcome:** Adherence (compare actual activities against the planned ones) **Methods:** logs, reports, chart review, observations	• guiding coalition • environment and policy assessment • staff and supervisor education • individualized walking goals • ongoing process evaluation and feedback	• Most residents were provided walking activities 60% to 90% of the planned time over a 20-week period • Average adherence to documenting the activity: 79%
**(B) organizational capacity-building interventions to promote and maintain the mobility of nursing home residents**				
Kuk et al. 2017 [57]	Activity innovation	**Aim:** to evaluate the feasibility of the TIP Toolbox to further improve the Toolbox in terms of end-user needs **Study design:** feasibility study with a mixed-methods design **Implementation outcome:** fidelity, dose, context, satisfaction, complexity, adaptations **Methods:** documentation analysis, questionnaires, telephone interviews, participant observations, and focus group interviews	Translating Innovations into Practice-toolbox (TIP-toolbox): implementation approach based on the “Implementation of Change Model” by Grol et al. [66] 1. formulating a proposal for change in practice including clear targets 2. assessing the nursing staff performance and existing barriers and formulating specific targets for change 3. selecting and tailoring a set of strategies together with nursing staff 4. planning the implementation process 5. integrating improvement within the normal practice routines 6. evaluating the plan Additional tools • MAINtAIn questionnaire to assess the extent to which nursing staff promote functional activity among residents and the perceived barriers and facilitators • excel-based analysis tool • overview of strategies • template implementation plan • example implementation plan • example of an innovation	• Most registered nurses completed all 6 steps of the implementation plan • The registered nurses conducted most steps according to the plan • Fidelity was affected the registered nurses’ difficulty in formulating SMART goals and a high time requirement for some steps • The registered nurses suggested several adaptations aimed at improving cooperation with others and increasing the feeling of support • Most registered nurses were satisfied with the TIP-toolbox and considered themselves capable of performing the steps, but some considered it somewhat complex and described different difficulties
Projekt ExMo Görres et al. 2016 [62]	National expert standard “Maintenance and promotion of mobility in care”	**Aim:** to evaluate the exemplary implementation of the draft expert standard “Maintenance and promotion of mobility in care” **Study design:** cluster randomized trial & observational study **Implementation outcome:** no information **Methods:** no information	In both intervention groups: • implementation materials (handbook, including a template for the documentation of the implementation process and an audit tool) • implementation strategies (e.g., formation of a project group, needs assessment, kick-offs, additional educational sessions)	Based on the results the authors conclude that the expert standard is feasible for practice use

### Factors influencing implementation

All 16 of the included studies described factors influencing the implementation of mobility interventions for NH residents. In total, we identified 32 of the 37 influencing factors of the CFIR (Table 4)[29]. The five most frequent influencing factors were *available resources* (*n* = 14)[43, 44, 46–51, 53, 56, 58–60, 62], *access to knowledge and information* (*n* = 12)[43, 44, 46–48, 53, 56, 58–62], *patient needs and resources* (*n* = 10)[43, 47–50, 52, 53, 56, 58, 59, 62], *knowledge and beliefs about the intervention* (*n* = 10)[43, 44, 46, 48, 49, 53, 56–58, 62] and *compatibility* (*n* = 9)[43, 48–50, 53, 58–60, 62].

**Table 4:** Overview of the influencing factors identified within each study

Relevant categories of the CFIR	(A) direct interventions to promote and maintain the mobility of nursing home residents	[49]	[50]	[48]	[47]	[44]	[51]	[46]	[43, 45]	(B) organizational capacity-building interventions to promote and maintain the mobility of nursing home residents	[61]	[62]	[60]	[57]	[53]	[59]	(A) direct interventions & (B) organizational capacity-building interventions to promote and maintain the mobility of nursing home residents	[52, 56]	[58]	*N*
*Intervention characteristics*																				
Evidence strength & quality		*x*	*x*	*x*				*x*	*x*				*x*			*x*		*x*		*8*
Relative advantage				*x*					*x*											*2*
Adaptability		*x*	*x*	*x*								*x*								*4*
Complexity				*x*				*x*				*x*							*x*	*4*
Design quality & packaging			*x*	*x*				*x*	*x*			*x*						*x*	*x*	*7*
*Outer setting*																				
Patient needs & resources		*x*	*x*	*x*	*x*				*x*			*x*			*x*	*x*		*x*	*x*	*10*
Cosmopolitanism												*x*								*1*
External policy & incentives												*x*								*1*
*Inner setting*																				
Structural characteristics						*x*		*x*	*x*			*x*			*x*			*x*		*6*
Networks & Communications				*x*			*x*	*x*				*x*	*x*	*x*	*x*					*7*
Culture							*x*				*x*	*x*		*x*	*x*	*x*		*x*	*x*	*8*
*Implementation climate*																				
Learning climate				*x*		*x*			*x*					*x*		*x*				5
Goals and feedback																*x*				1
Organizational incentives & rewards																		x		1
Relative priority				*x*	*x*				*x*		*x*							x	*x*	6
Compatibility		*x*	*x*	*x*					*x*			*x*	*x*		*x*	*x*			*x*	9
Tension for change					*x*															1
*Readiness for implementation*																				
Access to knowledge & information				*x*	*x*	*x*		*x*	*x*		*x*	*x*	*x*		*x*	*x*		*x*	*x*	*12*
Available resources		*x*	*x*	*x*	*x*	*x*	*x*	*x*	*x*			*x*	*x*		*x*	*x*		*x*	*x*	*14*
Leadership engagement					*x*	*x*	*x*		*x*		*x*			*x*		*x*		*x*		*8*
*Characteristics of individuals*																				
Knowledge & beliefs about the intervention		*x*		*x*		*x*		*x*	*x*			*x*		*x*	*x*			*x*	*x*	*10*
Self-efficacy					*x*						*x*	*x*			*x*				*x*	*5*
Individual stage of change																		*x*	*x*	*2*
Other personal attributes				*x*	*x*			*x*				*x*		*x*	*x*			*x*	*x*	*8*
*Process*																				
Planning												*x*								1
*Engaging*			*x*	*x*		*x*						*x*						x		5
Opinion leaders												*x*								1
Formally appointed internal implementation leaders				*x*								*x*				*x*				3
Champions					*x*															1
External change agents																*x*				1
Executing																		x		1
Reflecting & evaluating					*x*		*x*													2

*Available resources,* such as time [43, 44, 46, 47, 49, 53, 56, 60, 62] staff [43, 46, 48–51, 56, 58, 60, 62], environment [46, 49, 50, 56, 58, 62], (exercise) equipment [50, 53, 58, 59, 62], aids and trainings [53, 62], were described as the most frequent factors influencing implementation. In addition, *access to knowledge and information* related to sufficient information and training regarding the intervention [43, 47, 53, 56, 58, 59], transparency about the implementation project [43, 48, 58, 61], opportunity to attend trainings (e.g., shift arrangement, staff absence, vacation, lack of information) [56, 61, 62], different types [62] and multiple of training [46, 60], access for the whole team [61] and (enough) trained staff [58, 62] were mentioned as influencing factors. Influencing factors related to the residents (*patient needs and resources*) were motivation of the resident [43, 47, 48, 53, 62], resident compliance [49, 62], willingness to participate [43, 48, 53, 58], attitude and expectations toward the intervention and mobility [53, 58, 62], cognitive and physical abilities [43, 47–50, 53, 56, 58, 59, 62], health status (including pain and fatigue)[43, 49, 50, 52, 53, 58, 62] and social engagement [52]. Additionally, the persons present during the exercise (e.g., nurses, leaders, relatives)[47], the resident’s guidance and support [47, 53, 62], and responses to residents’ needs (e.g., giving time, simple commands, control, and verbal encouragement)[53] were reported. In relation to the residents, their relatives and relatives’ knowledge and expectations regarding the intervention and mobility [53, 59, 62], and their uncertainty [62], motivation [62], cooperation [47, 62] and involvement [47, 59, 62] were also mentioned as relevant factors. In addition, the knowledge and beliefs about the intervention of the individuals involved in the implementation were described frequently as factors that influenced implementation. In this regard, knowledge about the intervention and its benefits [43, 46, 49, 53, 57, 58, 62], expectations related to outcomes and workload [48, 53, 56, 58, 62], roles and task understanding [49], and attitudes toward the intervention [48, 56, 62] were reported. Another frequently mentioned influencing factor was the *compatibility* of the intervention with existing care/practice routines and workflows [58, 62], organizational structures [62] and organizational culture [48, 53], staff’s perceived risks [43, 49, 50, 53, 60, 62], resident rights [59] and other projects [62].

## Discussion

In this scoping review, we identified 16 studies [43–53, 56–62] that provided evidence on the implementation of interventions to promote or maintain the functional mobility of NH residents. Most of these studies presented evidence regarding factors influencing such implementation processes [43–53, 56–62], but the scope and depth of evidence varied between the studies. Studies systematically covering a broad range of different implementation aspects were mostly missing and were most likely to be found among those few studies that provide evidence on implementation strategies [42, 44–46, 51, 57, 62]. However, even among these studies, only the research program on the sit-to-stand activity examined implementation aspects with a successively developed step-by-step approach and with a long-term perspective considering study results from the previous steps to develop tailored implementation strategies [42, 45, 46]. This may reflect the lack of systematic approaches to designing implementation research [67], which was also underlined by a scoping review by Yang et al. 2020 [68]. In their review, the authors included RCTs on the effectiveness and implementation of recreational therapy programs to enhance functional mobility. Even though they were able to derive some evidence on implementation issues, the included RCTs mainly focused on intervention effectiveness.

Overall, it can be stated that the implementation of mobility interventions for NH residents has thus far been insufficiently investigated. Nevertheless, this review brings together the evidence available to date on this topic and thus provides valuable indications for the successful implementation of such interventions.

### Implementation strategies

Those studies that examined implementation strategies showed a range of different approaches. The study program of Slaughter et al. [42, 43, 45, 46, 51, 54, 55] referred to a simple direct mobility intervention and examined discrete implementation strategies [69], while the other studies examined either more complex organizational capacity building interventions[57, 62] or more complex implementation approaches [44, 57, 62].

Reminder systems, as systematically investigated by Slaughter et al. [45, 46], are common and listed in different taxonomies of implementation strategies (e.g. [69–71]). The findings that Slaughter et al. [45] reported have been supported by other studies. Cheung et al. [72] concluded from an overview of 35 systematic reviews that reminder systems are effective in improving healthcare professionals' behavior and that they are more likely to be effective if they are tailored to the respective care setting. In this regard, the literature describes very different types of reminders, ranging from very simple to highly complex formats [72]. Thus, the systematic approach described by Slaughter et al. [45, 46] to investigate different reminder systems meets these requirements.

Görres & Rothgang [62] suggested that that the combination of education sessions and accompanying implementation strategies was a successful strategy for the implementation of the national expert standard “Maintenance and promotion of mobility in care”. Generally, educational meetings are considered to be established in routine care and effective in improving professional practice [69, 73], but Forsetlund et al. [73] pointed out that "educational meetings alone are not likely to be effective for changing complex behaviours" (p. 1). Thus, the combination of educational sessions and additional implementation interventions as used by Görres & Rothgang [62] seems promising.

Often, not a single implementation strategy but rather multiple, more complex implementation strategies are used. For example, Powell et al. [69] understood their compilation of implementation strategies as a "list of discrete strategies that can serve as ‘building blocks’ for constructing multifaceted, multilevel implementation strategies" (p. 7). In line with this, Slaughter et al. [42, 43, 45, 51] also combined reminder systems with other strategies (e.g., audit-and-feedback intervention, informal walkabouts) and conclude this to be supportive for implementation success.

For the design of multifaceted, multilevel implementation strategies, it is recommended that they fit the respective implementation context [74, 75]. However, such tailoring of implementation strategies is particularly challenging [30]. In particular, the consideration of context-specific implementation determinants seems crucial for success [75, 76]. Accordingly, the approaches described in the studies by Kuk et al. [57] and Kazana & Pencak Murphy [44] are of interest, since both represent a comprehensive implementation approach focused on organizational development. In particular, the TIP-toolbox by Kuk et al. [57] represents a systematic approach for developing a tailored implementation strategy. The TIP-toolbox not only considers barriers and facilitators for the implementation but also represents a theory-driven and step-by-step approach that is guided by the implementation of change model [66]. This can be understood as a key success factor since theoretical framing is reasonable for the development and application of tailored implementation strategies [69, 77]. Additionally, Kazana & Pencak Murphy [44] considered barriers and facilitators within their approach.

In summary, the studies investigated a spectrum of promising implementation approaches. However, only the research program on the sit-to-stand activity showed a step-by-step and long-term approach.

### Influencing factors

All of the included studies reported influencing factors of the implementation of mobility interventions for NH residents. These influencing factors relate to the process of implementing mobility interventions. In this respect, the findings of our scoping review indicate which facilitating and inhibiting factors need to be considered when implementing mobility interventions into real-life settings. However, based on the findings, no statements can be made regarding the impact and causal connections of the mobility interventions applied in the identified studies.

Altogether a broad range of influencing factors was identified, which included almost all influencing factors that the CFIR comprises. Available resources, access to knowledge and information, patient needs and resources, knowledge and beliefs about the intervention and compatibility were identified as the most frequently reported influencing factors. Reviews addressing the implementation of other interventions, such as complex interventions in general, fall prevention or guidelines in NHs, have identified similar influencing factors [78–81]. In particular, lack of time, staffing ratio, missing equipment and training have been described as barriers [78–81] to the implementation of various interventions in NHs, and they represent a vital challenge of the conditions in this care setting. Abilities, attitudes, expectations of residents and the influence of relatives have also been described as important influencing factors in other reviews [79–81]. Implementation strategies such as resident and involvement of the relatives, knowledge transfer, and tailoring of interventions can be used to address this kind of factor that influences implementation. The matching tool developed by Waltz et al. [75] could – for example – be used to specifically address known barriers with suitable implementation strategies.

Regarding the different types of interventions (direct (A) vs. organizational capacity-building (B) interventions), no major differences in the influencing factors could be identified. The five most important influencing factors were reported with somewhat equal frequency relative to both types of interventions (A and B). Different influencing factors of the CFIR inner setting domain and its networks and communication and culture constructs were more often identified as influencing factors for the capacity-building interventions than for the direct interventions. Conversely, more influencing factors related to the CFIR domain characteristics of the intervention were identified for the direct interventions.

### Limitations

The scoping review had some limitations. The concept of organizational capacity-building interventions lacks an internationally established definition. Since the term “Verhältnisprävention” covers this concept for the German context, we built our search strategy B based on the definition of this term [82, 83]. However, an internationally accepted definition might have led to other aspects to be considered. Furthermore, we only included studies in English and German, and no librarian was involved in the development of the search strategies. However, the researchers involved had both subject-specific and methodological expertise in conducting reviews. Despite the limitations just described, we were able to generate valuable findings. Above all, the systematic approach based on proven standards [32] and the ongoing exchange within the research team contributed to this.

## Conclusions

The results of the review provide an overview of the currently rather limited evidence on the implementation of interventions to promote and maintain the functional mobility of NH residents. In particular, there have been few studies examining implementation strategies. However, these studies provide some promising approaches that can serve as a starting point for further research. Studies that evaluate discrete implementation strategies for direct mobility intervention (e.g., the sit-to-stand activity) as well as studies that further develop multifaceted, multicomponent implementation approaches (e.g., the TIP-toolbox) with a focus on complex interventions that also include capacity-building components are recommended here. In contrast, many of the studies reported influencing factors of the implementation. According to our findings, the implementation of mobility interventions especially required sufficient resources, access to knowledge and information for all staff, and consideration of the needs and resources of residents and their relatives. These findings can be considered in practice and research for the development of tailored strategies for the implementation of mobility interventions. Furthermore, the identified indications of differences between (A) direct and (B) capacity-building interventions might be considered in the process of developing tailored implementation strategies. However, further research is needed on this topic.

Finally, it is important to emphasize that the impact of the review’s findings extends beyond intervention studies in nursing home settings alone, and therefore hold relevance and value for various types of research in nursing homes and other geriatric care settings. They can provide guidance and insights for researchers and practitioners exploring different aspects of nursing home and geriatric care, including e.g., practice and quality improvement initiatives, resident well-being, or policy development.

### Supplementary Information

**Additional file 1. ** 

**Additional file 2.** 

**Additional file 3.** 

## Data Availability

The datasets used and/or analyzed during the current study are available from the corresponding author on reasonable request.

## Abbreviations

BZgA: Bundeszentrale für gesundheitliche Aufklärung
CFIR: Consolidated Framework for Implementation Research
NH: Nursing home
PCC mnemonic: Population, Concept of interest, Context mnemonic
PRISMA-ScR: Preferred Reporting Items for Systematic reviews and Meta-Analyses extension for Scoping Reviews
TIP-toolbox: Translating Innovations into Practice-toolbox

## References

[1] Cruz-Jimenez M (2017). Normal changes in gait and mobility problems in the elderly. Phys Med Rehabil Clin N Am.

[2] Diem SJ, Lui LY, Langsetmo L, Taylor B, Cawthon PM, Cauley JA (2018). Effects of mobility and cognition on maintenance of independence and survival among women in late life. J Gerontol A Biol Sci Med Sci.

[3] Goins RT, Jones J, Schure M, Rosenberg DE, Phelan EA, Dodson S (2015). Older adults' perceptions of mobility: a metasynthesis of qualitative studies. Gerontologist.

[4] Portegijs E, Rantakokko M, Viljanen A, Sipilä S, Rantanen T. Is frailty associated with life-space mobility and perceived autonomy in participation outdoors? A longitudinal study. Age Ageing. 2016;45(4):550-3 10.1093/ageing/afw072 27126330

[5] Rosso AL, Taylor JA, Tabb LP, Michael YL (2013). Mobility, disability, and social engagement in older adults. J Aging Health.

[6] Sundar V, Brucker DL, Pollack MA, Chang H (2016). Community and social participation among adults with mobility impairments: a mixed methods study. Disabil Health J.

[7] Trombetti A, Reid KF, Hars M, Herrmann FR, Pasha E, Phillips EM (2016). Age-associated declines in muscle mass, strength, power, and physical performance: impact on fear of falling and quality of life. Osteoporos Int.

[8] Groessl EJ, Kaplan RM, Rejeski WJ, Katula JA, King AC, Frierson G (2007). Health-related quality of life in older adults at risk for disability. Am J Prev Med.

[9] Stubbs B, Schofield P, Patchay S (2016). Mobility limitations and fall-related factors contribute to the reduced health-related quality of life in older adults with chronic musculoskeletal pain. Pain Pract.

[10] Davis JC, Best JR, Bryan S, Li LC, Hsu CL, Gomez C (2015). Mobility is a key predictor of change in well-being among older adults who experience falls: evidence from the vancouver falls prevention clinic cohort. Arch Phys Med Rehabil.

[11] Fagerström C, Borglin G (2010). Mobility, functional ability and health-related quality of life among people of 60 years or older. Aging Clin Exp Res.

[12] Musich S, Wang SS, Ruiz J, Hawkins K, Wicker E (2018). The impact of mobility limitations on health outcomes among older adults. Geriatr Nurs.

[13] Hennessy S, Kurichi JE, Pan Q, Streim JE, Bogner HR, Xie D (2015). Disability stage is an independent risk factor for mortality in medicare beneficiaries aged 65 years and older. PM&R.

[14] Hardy SE, Kang Y, Studenski SA, Degenholtz HB (2011). Ability to walk 1/4 mile predicts subsequent disability, mortality, and health care costs. J Gen Intern Med.

[15] Mänty M, Heinonen A, Viljanen A, Pajala S, Koskenvuo M, Kaprio J (2010). Self-reported preclinical mobility limitation and fall history as predictors of future falls in older women: prospective cohort study. Osteoporos Int.

[16] Deutsches Netzwerk für Qualitätsentwicklung in der Pflege (DNQP). Expertenstandard nach § 113a SGB XI Erhaltung und Förderung der Mobilität in der Pflege - Abschlussbericht. Osnabrück; 2014.

[17] Masciocchi E, Maltais M, Rolland Y, Vellas B, de Souto BP (2019). Time effects on physical performance in older adults in nursing home: a narrative review. J Nutr Health Aging.

[18] Wingenfeld K, Schröder D, Willert J, Bender B. Ergebnisse der Literaturanalyse zur Aktualisierung des Entwurfs des Expertenstandards „Erhaltung und Förderung der Mobilität in der Pflege“ Institut für Pflegewissenschaft an der Universität Bielefeld 2020.

[19] Deutsches Netzwerk für Qualitätsentwicklung in der Pflege (DNQP). Konsultationsfassung zum Expertenstandard nach § 113a SGB XI „Erhaltung und Förderung der Mobilität in der Pflege“ - Aktualisierung 2020 - Präambel, Expertenstandard und Kommentierung. 2020.

[20] Rommerskirch-Manietta M, Braunwarth JI, Quasdorf T, Manietta C, Rodrigues-Recchia D, Reuther S, et al. Organizational Capacity Building in Nursing Facilities to Promote Resident Mobility: A Systematic Review. J Am Med Dir Assoc. 2021;22(12):2408–24 e12. 10.1016/j.jamda.2021.09.017 34653383

[21] Kovács E, Sztruhár Jónásné I, Karóczi CK, Korpos A, Gondos T (2013). Effects of a multimodal exercise program on balance, functional mobility and fall risk in older adults with cognitive impairment: a randomized controlled single-blind study. Eur J Phys Rehabil Med.

[22] Telenius EW, Engedal K, Bergland A (2015). Effect of a high-intensity exercise program on physical function and mental health in nursing home residents with dementia: an assessor blinded randomized controlled trial. PLoS One.

[23] Arrieta H, Rezola-Pardo C, Zarrazquin I, Echeverria I, Yanguas JJ, Iturburu M (2018). A multicomponent exercise program improves physical function in long-term nursing home residents: A randomized controlled trial. Exp Gerontol.

[24] Craig P, Dieppe P, Macintyre S, Michie S, Nazareth I, Petticrew M (2008). Developing and evaluating complex interventions: new guidance.

[25] Moore GF, Audrey S, Barker M, Bond L, Bonell C, Hardeman W, et al. Process evaluation of complex interventions: Medical Research Council guidance. BMJ. 2015;350. 10.1136/bmj.h1258 PMC4366184 25791983

[26] Moore GF, Audrey S, Barker M, Bond L, Bonell C, Hardeman W, et al. Process evaluation of complex interventions - UK Medical Research Council (MRC) guidance. London: MRC Population Health Sciences Research Network; 2014 05/06/2020.

[27] Vernooij-Dassen M, Moniz-Cook E (2014). Raising the standard of applied dementia care research: addressing the implementation error. Aging Ment Health.

[28] Chaudoir SR, Dugan AG, Barr CH (2013). Measuring factors affecting implementation of health innovations: a systematic review of structural, organizational, provider, patient, and innovation level measures. Implement Sci.

[29] Damschroder LJ, Aron DC, Keith RE, Kirsh SR, Alexander JA, Lowery JC (2009). Fostering implementation of health services research findings into practice: a consolidated framework for advancing implementation science. Implement Sci.

[30] Powell BJ, Beidas RS, Lewis CC, Aarons GA, McMillen JC, Proctor EK (2017). Methods to improve the selection and tailoring of implementation strategies. J Behav Health Serv Res.

[31] Peters M, Godfrey C, McInerney P, Munn Z, Tricco A, Khalil H. Chapter 11: Scoping Reviews (2020 version). In: Aromataris E, Munn Z, editors. JBI Manual for Evidence Synthesis: JBI; 2020.

[32] Peters MDJ, Marnie C, Tricco AC, Pollock D, Munn Z, Alexander L (2020). Updated methodological guidance for the conduct of scoping reviews. JBI Evidence Synthesis.

[33] Tricco AC, Lillie E, Zarin W, O'Brien KK, Colquhoun H, Levac D (2018). PRISMA extension for scoping reviews (PRISMA-ScR): checklist and explanation. Ann Intern Med.

[34] McGowan J, Sampson M, Salzwedel DM, Cogo E, Foerster V, Lefebvre C (2016). PRESS peer review of electronic search strategies: 2015 guideline statement. J Clin Epidemiol.

[35] Nordhausen T, Hirt J. RefHunter - Manual zur Literaturrecherche in Fachdatenbanken - Version 5.0: Martin-Luther-Universität Halle-Wittenberg; OST (ehemals FHS St. Gallen); 2020.

[36] Peters MDJ, Godfrey C, McInerney P, Munn Z, Tricco AC, Khalil H. Chapter 11: Scoping Reviews (2020 version). In: Aromataris E, Munn Z, editors. JBI Manual for Evidence Synthesis 2020.

[37] De Bock F, Geene R, Hoffmann W, Stang A. Vorrang für Verhältnisprävention - Handreichung aus der Steuerungsgruppe des Zukunftsforums Public Health für alle mit Prävention in Praxis und Politik befassten Akteure. Berlin; 2017. Accessed 28 Aug 2023.

[38] Potter C, Brough R (2004). Systemic capacity building: a hierarchy of needs. Health Policy Plan.

[39] Covidence. Systematic review software, Veritas Health Innovation, Melbourne, Australia. 2020. Available from: www.covidence.org. Accessed 28 Aug 2023.

[40] Elo S, Kyngas H (2008). The qualitative content analysis process. J Adv Nurs.

[41] VERBI Software. MAXQDA. Software für qualitative Datenanalyse. 2020.2.0 ed. Berlin, Deutschland: Consult. Sozialforschung GmbH; 1989–2021.

[42] Tworek KB, Ickert C, Bakal J, Eliasziw M, Wagg AS, Jones CA, et al. Examining the Impact of Knowledge Translation Interventions on Uptake of Evidence-Based Practices by Care Aides in Continuing Care. Worldviews Evid Based Nurs. 2019. 10.1111/wvn.12344 30701658

[43] Kagwa SA, Boström A-M, Ickert C, Slaughter SE. Optimising mobility through the sit-to-stand activity for older people living in residential care facilities: a qualitative interview study of healthcare aide experiences. Int J Older People Nurs. 2018;13:e12169. 10.1111/opn.12169. 10.1111/opn.12169 28971588

[44] Kazana I, Pencak MM (2018). Implementing a patient-centered walking program for residents in long-term care: A quality improvement project. J Am Assoc Nurse Pract.

[45] Slaughter SE, Eliasziw M, Ickert C, Jones CA, Estabrooks CA, Wagg AS (2020). Effectiveness of reminders to sustain practice change among direct care providers in residential care facilities: a cluster randomized controlled trial. Implement Sci.

[46] Slaughter SE, Bampton E, Erin DF, Ickert C, Wagg AS, Allyson Jones C (2018). Knowledge translation interventions to sustain direct care provider behaviour change in long-term care: A process evaluation. J Eval Clin Pract.

[47] Juul A, Wilding R, Baldassar L. The best day of the week: new technology enhancing quality of life in a care home. Int J Environ Res Public Health. 2019;16(6):1000. 10.3390/ijerph16061000. 10.3390/ijerph16061000 PMC6466428 30893945

[48] Horn A, Kleina T, Schaeffer D (2019). Success factors and obstacles in the implementation of the "Lubeck Worlds of Movement Model" in inpatient care facilities-results of a scientific evaluation. Bundesgesundheitsblatt Gesundheitsforschung Gesundheitsschutz.

[49] Brett L, Traynor V, Stapley P, Meedya S (2018). Exercise and dementia in nursing homes: views of staff and family carers. J Aging Phys Act.

[50] Gomaa YS, Slade SC, Tamplin J, Wittwer JE, Gray R, Blackberry I (2020). Therapeutic dancing for frail older people in residential aged care: a thematic analysis of barriers and facilitators to implementation. Int J Aging Hum Dev.

[51] Slaughter SE, Estabrooks CA (2013). Optimizing the mobility of residents with dementia: a pilot study promoting healthcare aide uptake of a simple mobility innovation in diverse nursing home settings. BMC Geriatr.

[52] Finnegan S, Bruce J, Lamb SE, Griffiths F (2015). Predictors of attendance to group exercise: a cohort study of older adults in long-term care facilities. BMC Geriatr.

[53] Resnick B, Simpson M, Galik E, Bercovitz A, Gruber-Baldini AL, Zimmerman S (2006). Making a difference: nursing assistants' perspectives of restorative care nursing. Rehabil Nurs.

[54] Slaughter SE, Estabrooks CA, Jones CA, Wagg AS (2011). Mobility of Vulnerable Elders (MOVE): study protocol to evaluate the implementation and outcomes of a mobility intervention in long-term care facilities. BMC Geriatr..

[55] Slaughter SE, Estabrooks CA, Jones CA, Wagg AS, Eliasziw M (2013). Sustaining Transfers through Affordable Research Translation (START): study protocol to assess knowledge translation interventions in continuing care settings. Trials..

[56] Ellard DR, Thorogood M, Underwood M (2014). Whole home exercise intervention for depression in older care home residents (the OPERA study): a process evaluation. BMC Med..

[57] Kuk NO, Bours GJJW, Hamers JPH, Kempen GIJM, Zijlstra GAR (2017). Feasibility of the Translating Innovations into Practice-toolbox (TIP-toolbox): a mixed-methods study for implementing activity innovations in nursing homes. Geriatr Nurs.

[58] Turpie L, Whitelaw S, Topping C (2017). Physical activity promotion in care homes. Working with Older People.

[59] Resnick B, Petzer-Aboff I, Galik E, Russ K, Cayo J, Simpson M (2008). Barriers and benefits to implementing a restorative care intervention in nursing homes. J Am Med Dir Assoc.

[60] Henskens M, Nauta IM, Scherder EJA, Oosterveld FGJ, Vrijkotte S (2017). Implementation and effects of Movement-oriented Restorative Care in a nursing home - a quasi-experimental study. BMC Geriatr.

[61] den Ouden M, Zwakhalen SMG, Meijers JMM, Bleijlevens MHC, Hamers JPH (2019). Feasibility of DAIly NURSE: A nursing intervention to change nursing staff behaviour towards encouraging residents' daily activities and independence in the nursing home. J Clin Nurs.

[62] Görres S, Rothgang H (2016). Modellhafte Implementierung des Expertenstandard-Entwurfs "Erhaltung und Förderung der Mobilität in der Pflege".

[63] Page MJ, McKenzie JE, Bossuyt PM, Boutron I, Hoffmann TC, Mulrow CD (2021). The PRISMA 2020 statement: an updated guideline for reporting systematic reviews. BMJ.

[64] Saunders RP, Evans MH, Joshi P (2005). Developing a process-evaluation plan for assessing health promotion program implementation: a how-to guide. Health Promot Pract.

[65] Kuk NO, Zijlstra GAR, Bours G, Hamers JPH, Kempen G (2016). Development and usability of the MAINtAIN, an inventory assessing nursing staff behavior to optimize and maintain functional activity among nursing home residents: a mixed-methods approach. BMC Health Serv Res.

[66] Grol R, Wensing M, Eccles M, Davis D. Improving Patient Care: The Implementation of Change in Health Care, Second Edition. 2013.

[67] Hull L, Goulding L, Khadjesari Z, Davis R, Healey A, Bakolis I (2019). Designing high-quality implementation research: development, application, feasibility and preliminary evaluation of the implementation science research development (ImpRes) tool and guide. Implement Sci.

[68] Yang Y, van Schooten KS, McKay HA, Sims-Gould J, Hoang RA, Robinovitch SN (2020). Recreational Therapy to Promote Mobility in Long-Term Care. J Aging Phys Act.

[69] Powell BJ, Waltz TJ, Chinman MJ, Damschroder LJ, Smith JL, Matthieu MM (2015). A refined compilation of implementation strategies: results from the Expert Recommendations for Implementing Change (ERIC) project. Implement Sci.

[70] Leeman J, Baernholdt M, Sandelowski M (2007). Developing a theory-based taxonomy of methods for implementing change in practice. J Adv Nurs.

[71] Waltz TJ, Powell BJ, Matthieu MM, Damschroder LJ, Chinman MJ, Smith JL (2015). Use of concept mapping to characterize relationships among implementation strategies and assess their feasibility and importance: results from the Expert Recommendations for Implementing Change (ERIC) study. Implement Sci.

[72] Cheung A, Weir M, Mayhew A, Kozloff N, Brown K, Grimshaw J. Overview of systematic reviews of the effectiveness of reminders in improving healthcare professional behavior. Syst Rev. 2012;1:36-. 10.1186/2046-4053-1-36 PMC3503870 22898173

[73] Forsetlund L, Bjorndal A, Rashidian A, Jamtvedt G, O'Brien MA, Wolf F, et al. Continuing education meetings and workshops: effects on professional practice and health care outcomes. Cochrane Database Syst Rev. 2009(2):CD003030. 10.1002/14651858.CD003030.pub2 PMC7138253 19370580

[74] Baker R, Camosso‐Stefinovic J, Gillies C, Shaw EJ, Cheater F, Flottorp S, et al. Tailored interventions to overcome identified barriers to change: effects on professional practice and health care outcomes. Cochrane Database Syst Rev. 2010;(3). Art. No.: CD005470. 10.1002/14651858.CD005470.pub2. Accessed 18 Sept 2023. 10.1002/14651858.CD005470.pub2 PMC4164371 20238340

[75] Waltz TJ, Powell BJ, Fernandez ME, Abadie B, Damschroder LJ (2019). Choosing implementation strategies to address contextual barriers: diversity in recommendations and future directions. Implement Sci.

[76] Grimshaw JM, Eccles MP, Lavis JN, Hill SJ, Squires JE (2012). Knowledge translation of research findings. Implement Sci.

[77] Proctor EK, Brownson RC, Brownson RC, Colditz GA, Proctor EK (2012). Measurement issues in dissemination and implementation research. Dissemination and implementation research in health - translating science to practice.

[78] Groot Kormelinck CM, Janus SIM, Smalbrugge M, Gerritsen DL, Zuidema SU (2021). Systematic review on barriers and facilitators of complex interventions for residents with dementia in long-term care. Int Psychogeriatr.

[79] Korall AM, Feldman F, Scott VJ, Wasdell M, Gillan R, Ross D (2015). Facilitators of and barriers to hip protector acceptance and adherence in long-term care facilities: a systematic review. J Am Med Dir Assoc.

[80] McArthur C, Bai Y, Hewston P, Giangregorio L, Straus S, Papaioannou A (2021). Barriers and facilitators to implementing evidence-based guidelines in long-term care: a qualitative evidence synthesis. Implement Sci..

[81] Vlaeyen E, Stas J, Leysens G, Van der Elst E, Janssens E, Dejaeger E, Dobbels F, Milisen K (2017). Implementation of fall prevention in residential care facilities: a systematic review of barriers and facilitators. Int J Nurs Stud..

[82] Bundesministerium für Gesundheit (BMG). 2022. Available from: https://www.bundesgesundheitsministerium.de/service/begriffe-von-a-z/p/praevention.html#:~:text=Die%20Verh%C3%A4ltnispr%C3%A4vention%20ber%C3%BCcksichtigt%20unter%20anderem,das%20Einkommen%20und%20die%20Bildung. Accessed 18 Sept 2023.

[83] GKV-Spitzenverband. 2022. Available from: https://www.gkv-buendnis.de/glossar/?no_cache=1&filter=v&name=Verhaltens. Accessed 18 Sept 2023.

