# Supplementary material for: Implementation of interventions to maintain and promote the functional mobility of nursing home residents – a scoping review
Source: BMC Geriatr. 2023 Sep 26;23:600. doi: 10.1186/s12877-023-04213-5 (PMC10523713; doi:10.1186/s12877-023-04213-5)
Supplement: Supplementary file 2 — Additional file 2. [file 12877_2023_4213_MOESM2_ESM.docx]

**Supplementary Table 2: Search strategies for MEDLINE (via PubMed)**

|  | Direct interventions (A) | Organizational capacity building interventions (B) |
| --- | --- | --- |
| Context (C) = mobility intervention | #1 "Motor Activity"[Mesh]  #2 "Motor skills"[Mesh]  #3 "Locomotion"[Mesh]  #4 locomotion[TIAB]  #5 balance[TIAB]  #6 "Physical Fitness"[Mesh]  #7 physical[TIAB]  #8 function*[TIAB]  #9 Walking"[Mesh]  #10 walk*[TIAB]  #11 motor[TIAB]  #12 mobil*[TIAB]  #13 gait[TIAB]  #14 ambulat*[TIAB]  #15 moving[TIAB]  #16 movability [TIAB]  #17 OR/ #1-16  #18 prevention[TIAB]  #19 therapy[TIAB]  #20 exercise[TIAB]  #21 intervention*[TIAB]  #22 training[TIAB]  #23 activit*[TIAB]  #24 rehabilitation[TIAB]  #25 "Exercise"[Mesh]  #26 "Exercise Therapy"[Mesh]  #27 "Rehabilitation"[Mesh]  #28 or/#18-27  #29 #17 AND #28  #30 sport[TIAB]  #31 physiotherapy[TIAB]  #32 "Sports"[Mesh]  #33 or/#30-32  #34 #29 or #33 | #1 "physical activities" [Mesh]  #2 physical activities [TIAB]  #3 walk*[TIAB]  #4 walking [Mesh]  #5 activity [TIAB]  #6 ambulat*[TIAB]  #7 mobil*[TIAB]  #8 moving [TIAB]  #9 gait [TIAB]  #10 function [TIAB]  #11 physical function [TIAB]  #12 physical fitness [TIAB]  #13 physical fitness [Mesh]  #14 motor activities [Mesh]  #15 motor activities [TIAB]  #16 movability [TIAB]  #17 locomotion [Mesh]  #18 locomotion [TIAB]  #19 or/1-18  #20 architecture [Mesh]  #21 architecture [TIAB]  #22 environment [Mesh]  #23 environment* [TIAB]  #24 education [Mesh]  #25 education [TIAB]  #26 teaching [Mesh]  #27 teaching[TIAB]  #28 training[TIAB]  #29capability[TIAB]  #30 capacity building[TIAB]  #31 light*[TIAB]  #32 daylight[TIAB]  #33 workflow[TIAB]  #34 system[TIAB]  #35 change process[TIAB]  #36 work structure[TIAB]  #37 policy[TIAB]  #38 regulation[TIAB]  #39 staff*[TIAB]  #40 nursing staff[MeSH]  #41 walking way[TIAB]  #42 parkour[TIAB]  #43 garden[TIAB]  #44 floor*[TIAB]  #45 assistive technology[TIAB]  #46 assistive aids[TIAB]  #47 person-centred care[TIAB]  #48 person-centered care[TIAB]  #49 medication[TIAB]  #50 intervention[TIAB]  #51 prevention[TIAB]  #52 OR/#20-51  #53 #18 AND #52 |
| Concept (C) = implementation | #35 implement*[TI]  #36 health plan implementation[MeSH]  #37 "quality improvement*"[TIAB]  #38 quality improvement[MeSH]  #39 diffused[TIAB]  #40 diffusion[TIAB]  #41 diffusion of innovation[MeSH]  #42 "knowledge translation*"[TIAB]  #43 "knowledge exchange"[TIAB]  #44 "knowledge circulation"[TIAB]  #45 facilitators[TIAB]  #46 barriers[TIAB]  #47 "process evaluation*"[TIAB]  #48 "formative evaluation*"[TIAB]  #49 "summative evaluation*"[TIAB]  #50 "qualitative evaluation*"[TIAB]  #51 sustainability[TIAB]  #52 practicability[TIAB]  #53 feasibility[TIAB]  #54 fidelity[TIAB]  #55 maintenance[TIAB]  #56 adopt*[TIAB]  #57 OR/#35-56  #58 #34 AND #57 | #54 implement*[TI]  #55 health plan implementation[MeSH]  #56 "quality improvement*"[TIAB]  #57 quality improvement[MeSH]  #58 diffused[TIAB]  #59 diffusion[TIAB]  #60 diffusion of innovation[MeSH]  #61 "knowledge translation*"[TIAB]  #62 "knowledge exchange"[TIAB]  #63 "knowledge circulation"[TIAB]  #64 facilitators[TIAB]  #65 barriers[TIAB]  #66 "process evaluation*"[TIAB]  #67 "formative evaluation*"[TIAB]  #68 "summative evaluation*"[TIAB]  #69 "qualitative evaluation*"[TIAB]  #70 sustainability[TIAB]  #71 practicability[TIAB]  #72 feasibility[TIAB]  #73 fidelity[TIAB]  #74 maintenance[TIAB]  #75 adopt*[TIAB]  #76 OR/#54-75  #77 #53 AND #76 |
| Population(P) = long-term care | #59 long term care[MeSH Terms]  #60 residential facilities[MeSH Terms]  #61 skilled nursing facilities[MeSH]  #62 residential facilit*[TIAB]  #63 skilled nursing facilit*[TIAB]  #64 nursing home*[TIAB]  #65 homes for the aged[TIAB]  #66 care home*[TIAB]  #67 long term care[TIAB]  #68 short term care[TIAB]  #69 OR/#59-68  #70 #58 AND #69 | #78 long term care[MeSH Terms]  #79 residential facilities[MeSH Terms]  #80 skilled nursing facilities[MeSH]  #81 residential facilit*[TIAB]  #82 skilled nursing facilit*[TIAB]  #83 nursing home*[TIAB]  #84 homes for the aged[TIAB]  #85 care home*[TIAB]  #86 long term care[TIAB]  #87 short term care[TIAB]  #88 OR/#78-87  #89 #77 AND #88 |
