# Supplementary material for: Implementation of interventions to maintain and promote the functional mobility of nursing home residents – a scoping review
Source: BMC Geriatr. 2023 Sep 26;23:600. doi: 10.1186/s12877-023-04213-5 (PMC10523713; doi:10.1186/s12877-023-04213-5)
Supplement: Supplementary file 3 — Additional file 3. [file 12877_2023_4213_MOESM3_ESM.docx]

**Supplementary table 4:** **Coding categories** **for potential factors influencing the implementation processes (based on the Consolidated Framework for Implementation Research, CFIR^1^)**

| **Categories** | **Subcategories** |
| --- | --- |
| *Intervention  characteristics* | - Intervention source - Evidence strength and quality - Relative advantage - Adaptability - Trialability - Complexity - Design quality and packaging - Cost |
| *Outer setting* | - Patient needs and resources - Cosmopolitanism - Peer pressure - External policy and incentives |
| *Inner setting* | - Structural characteristics - Networks and communications - Culture - Implementation climate - Tension for change - Compatibility - Relative priority - Organizational incentives and rewards - Goals and feedback - Learning climate - Readiness for implementation - Leadership engagement - Available resources - Access to knowledge and information |
| *Characteristics of  individuals* | - Knowledge and beliefs about the intervention - Self-efficacy - Individual stage of change - Individual identification with organization - Other personal attributes |
| *Process* | - Planning - Engaging - Opinion leaders - Formally appointed internal implementation leaders - Champions - External change agents - Executing - Reflecting and Evaluating |

1. Damschroder, LJ, Aron, DC, Keith, RE, et al. Fostering implementation of health services research findings into practice: a consolidated framework for advancing implementation science. Implement Sci 2009;4:50.
